# Irrigation has more influence than fertilization on leaching water quality and the potential environmental risk in excessively fertilized vegetable soils

**DOI:** 10.1371/journal.pone.0204570

**Published:** 2018-09-27

**Authors:** Yang Li, Juanqi Li, Lihong Gao, Yongqiang Tian

**Affiliations:** 1 Beijing Key Laboratory of Growth and Developmental Regulation for Protected Vegetable Crops, China Agricultural University, Beijing, China; 2 College of Horticulture, Henan Agricultural University, Zhengzhou, China; Agroecological Institute, CHINA

## Abstract

Excessive fertilization is a common agricultural practice that often negatively influence soil and environmental quality in intensive vegetable production systems in China. To reduce negative effects of excessive fertilization, current studies generally focused on fertilizer management but not irrigation. In this study, we investigated the effects of fertilization and irrigation on soil properties, leaching water characteristics, plant growth, cucumber yield, irrigation water use efficiency (IWUE) and partial factor productivity of nitrogen (PFP_N_) in a double cropping system. The treatments included (i) conventional irrigation with conventional N fertilization (IcNc), (ii) optimal irrigation with conventional N fertilization (IoNc), (iii) conventional irrigation with optimal N fertilization (IcNo), and (iv) optimal irrigation with optimal N fertilization (IoNo). In general, fertilization merely influenced concentrations of nitrate (NO_3_^-^), phosphorus (P) and potassium (K), but did not affect most leaching water characteristics. In contrast, irrigation influenced pH, EC and concentrations of P, K, Ca, Mg, Na and Cu. Cumulative leached amounts of NO_3_^-^, P, K, Ca, Mg, Na, Fe, Cu and Zn were significantly decreased by optimal irrigation as compared to conventional irrigation under same fertilization conditions, but not by optimal fertilization as compared to conventional fertilization under same irrigation conditions. The leachate volume was strongly positively correlated with cumulative leached amounts of all tested elements, and these relationships were obviously influenced by irrigation but not fertilization. The IoNo treatment significantly increased both IWUE and PFP_N_ as compared to the IcNc treatment. However, the IcNo treatment only enhanced PFP_N_, while the IoNc treatment improved IWUE, when compared to the IcNc treatment. Our results suggested that irrigation has more influence than fertilization on leaching water quality and that the optimal irrigation combined with optimal fertilization was efficient in reducing the potential environmental risk caused by excessive fertilization in intensive vegetable production systems.

## Introduction

China’s population accounts for approximately 19% of the world’s population. However, China’s arable land accounts for only about 7% of the cultivated land in the world. To produce adequate food, since the early 1980s, Chinese agriculture has intensified greatly on a limited land area with large inputs of fertilizers, and has finally obtained relatively high crop yields on limited land [1]. Solar greenhouse is a common vegetable cultivation facility in China. Since solar greenhouse uses solar energy as the sole source of light and heat for vegetable production, it has developed rapidly in China [2], encompassing 4.0 million ha in 2015 [3]. Unfortunately, to obtain high crop yields, Chinese farmers often apply excessive fertilizers and irrigation water during crop production. For instance, in some greenhouse vegetable cultivation regions in China, annual irrigation rate was as high as 1000 mm, and fertilizer N apparent recovery efficiency was only 18–33% of applied N taken up by the vegetables. [4]. Consequently, redundant water and fertilizers can cause serious environmental problems, such as greenhouse gas emission, soil degradation, freshwater contamination, and natural resource consumption [5,6].

In recent years, efficient water and fertilizer managements have been increasingly applied in vegetable production in solar greenhouse. For instance, under moderate deficit irrigation (90% evapotranspiration (ET)) condition, a reduction of N fertilizer input did not decrease melon yield, but increased water and N fertilizer use efficiencies [7]. Similarly, Migliaccio et al. [8] reported water savings of 64–69% using tensiometers set at 10, 15 and 25 kPa compared with irrigation based on local schedule in papaya cultivation system in southern Florida.

Excessive fertilization is still common in solar greenhouse vegetable production in China [9,10]. However, farmers commonly use a large amount of fertilizers to avoid any possible negative effects on yield due to nutrient shortage and to ensure maximum yields of the marketable products [11] in solar greenhouse vegetable production. For instance, the annual nitrogen (N) input was > 3000 kg ha^-1^ in a solar greenhouse vegetable production system in China [9]. Obviously, this fertilization rate is too high to ensure not only fertilizer use efficiency, but also food safety. Due to excessive N fertilizer application, the fertilizer N recovery rate was less than 10% in most solar greenhouse vegetable production systems. This means that a large amount of N might leach into groundwater, enhancing the potential groundwater contamination. Indeed, a recent survey found that in solar greenhouse vegetable production systems, the nitrate concentration of the leachate ranged from 100 to 289 mg L^-1^, and exceeded the threshold of drinking water (50 mg L^-1^) recommended by the World Health Organization [12].

In addition to excessive fertilization, excessive irrigation is another common agricultural practice needed to be concerned in solar greenhouse vegetable production systems [10]. Compared with open-field crops, solar greenhouse vegetable crops often require more irrigation water input [13,14]. It seems reasonable to increase irrigation amount in solar greenhouse vegetable production because there is no precipitation in solar greenhouse and because plant transpiration rate is generally higher in greenhouse than in open field. However, since excessive fertilization can easily result in soil secondary salinization, which often un-benefits crop growth, excessive irrigation is commonly applied by farmers to reduce nutrient accumulation in top soils. Because of the lack of subsurface drainage water in greenhouse, excessive irrigation, combined with excessive fertilization, may easily lead to serious nutrient leaching and then groundwater contamination [15,16].

Leaching of N and P from agricultural soils, due to the excessive manure and synthetic fertilizer use, has been shown to contribute to the increased NO_3_^-^ concentrations and eutrophication of groundwater [17]. In addition to N and P, other nutrients, such as K, Ca, Mg, Na, Fe, Cu and Zn, may also be leached into groundwater under excessive irrigation conditions. To date, however, little information is available regarding the leaching loss of other nutrients (e.g. K, Ca, Mg, Na, Fe, Cu and Zn) under excessive fertilization conditions in solar greenhouses.

In addition to water and fertilizer management, soil physical conditions can also influence soil properties, nutrient leaching and crop yields. For instance, a previous study [18] reported that the amount of irrigation water and soil texture could interactively affect soil evapotranspiration, crop yield and crop water productivity in a cotton-wheat rotation system. The same study found that, with reducing irrigation water from 300 to 75 mm, the cotton yield was decreased by 17.7%, 58.2% and 74.0% in silt loam, sandy loam and loamy sand soils, respectively. This indicates that soils with different physical properties have different response to similar irrigation management. In addition to irrigation, fertilization may also show different effects on plant growth and crop yields under different soil physical conditions. For example, in an experiment related to maize production, urea application resulted in higher soil N availability and maize yields at clayey than at sandy soil types [19]. Moreover, nutrient leaching can also be influenced by soil types. Generally, nitrate concentration in upper groundwater is obvious lower in clay than in sandy soils [20]. Therefore, effects of soil types on water and fertilizer use efficiency should be considered to optimize the irrigation and fertilization.

Optimal irrigation and fertilization have been widely used to reduce water and fertilizer inputs without affecting crop yields [8] and to minimize water and nutrient losses due to leaching, evaporation and volatilization [21]. Optimal fertilization is a technique used to enhance fertilizer use efficiency according to the actual nutrient requirement of the plants, through applying appropriate rate and time of fertilization, fertilizer formulation, and the application method of fertilizer. With respect to optimal irrigation, it is a technique used to manage water supply for crops based on the actual water requirement of the plants, soil evapotranspiration and soil water content [8,22]. Traditionally, however, farmers often apply fertilizers and water based on experience or advice from other farmers without considering the actual nutrient and water requirement of the plants. As a result, under traditional fertilization and irrigation, the amounts of fertilizer and water are too high for crop to efficiently use.

Cucumber is a worldwide cultivated crop and China accounts for about 77% of the global production [23], it is also one of the major solar greenhouse vegetables in China. Both excessive fertilization and excessive irrigation are common agricultural practices in solar greenhouse cucumber production, causing imbalanced soil nutrients, increased nutrient leaching loss, and decreased nutrient and water use efficiencies. Thus, not only to alleviate environmental problems caused by excessive fertilization, but also to guarantee the crop production, farmers should be encouraged to reduce not only fertilizer use, but also irrigation water input. However, since most related researches focused on excessive fertilization [1,6], the importance of reducing irrigation water inputs has been ignored, at least partly. The preferential flow was the prerequisite for the leaching process, therefore, we hypothesize that irrigation has more influence than fertilization on nutrient leaching and leaching water quality in excessively fertilized soils, and that optimal irrigation is more efficient than optimal fertilization in reducing nutrient leaching in excessively fertilized soils. To test this hypothesis, in this study we conducted a solar greenhouse field experiment to investigate the effects of conventional fertilization and irrigation, and optimal fertilization and irrigation on soil water and nutrients, leaching water quality, plant growth, cucumber yield, irrigation water use efficiency (IWUE) and partial factor productivity of applied nitrogen (PFP_N_). To our knowledge, little information is available regarding leaching water quality including N, P, K, Ca, Mg, Na, Fe, Cu, and Zn in Chinese intensive greenhouse vegetable production systems. The aims of this study were to examine (1) whether nutrient leaching caused by excessive fertilization can be reduced by optimal irrigation, (2) how leaching water quality and plant growth may be affected by conventional excessive and/or optimal fertilization and irrigation, and (3) quantify the amount of nutrient elements in leaching water under both excessive (conventional) and optimal (recommended) irrigation and fertilization conditions.

## Materials and methods

### Site description and experimental design

The experiment was conducted in a solar greenhouse, covered with polyethylene film (ground area 70 m × 6 m) without supplementary lighting and heating, in Shunyi district, Beijing, China, from 2013 to 2014. The surface soil in the greenhouse (0–40 cm layer) had a pH (1:2.5 soil/water; *w*/*v*) of 6.83, an electrical conductivity (EC) (1:5 soil/water w/v) of 0.38 mS cm^-1^, a field capacity of 29% and a bulk density of 1.36 g cm^-3^, and contained 18.6 g kg^-1^ organic matter, 1.27 g kg^-1^ total nitrogen, 166.6 mg kg^-1^ mineral N, 193.48 mg kg^-1^ available phosphorus (P) and 357.8 mg kg^-1^ available potassium (K).

The experimental period comprised two growth cycles including the autumn-winter (AW) (from September 29 to January 20) and winter-spring (WS) (from March 1 to May 28) seasons. Cucumber seedlings (*Cucumis sativus* L. *cv*. Zhongnong No. 3) with two leaves were transplanted into soils by hand, with double rows of 90-cm row spacing and 30-cm plant spacing on the seedbed. Before transplanting, chicken manure (15 t ha^-1^; total N, total P, and total K contents were 33.5 g kg^−1^, 13.1 g kg^−1^ and 2.83 g kg^−1^, respectively) were incorporated into topsoils as basal fertilizer for two cropping seasons, and then were ploughed and harrowed.

Fertilization and irrigation were managed (Table 1) to create treatments as follows:

1. Conventional irrigation with conventional N fertilization (IcNc): Irrigation and N fetilization were applied at rates based on average levels used by greenhouse cucumber growers in the suburb of Beijing.
2. Optimal irrigation with conventional N fertilization (IoNc): Optimal irrigation was applied based on our previous study (65% and 95% of field capacity as the lower and upper limitations in AW season, and 75% and 95 of field capacity in WS season, respectively;), while N fertilization was applied at rates based on average levels used by greenhouse cucumber growers in the suburb of Beijing.
3. Conventional irrigation with optimal N fertilization (IcNo): Irrigation was applied at rates based on average levels used by greenhouse cucumber growers in the suburb of Beijing, while N fertilization was applied at rates based on N balance. Based on the N requirement for cucumber growth and N fertilizer recommendation, the total N rates applied by topdressing were 169.5 and 337.5 kg N ha^-1^ in the WS and AW season, respectively [24,25,26]. These total mineral N (N_min_) application rates were calculated using the method of soil N balance, where expected yield of solar greenhouse cucumber was 90 and 45 t ha^-1^ in the WS and AW seasons, respectively. The equation [27] was as follows:
  $$N_{recommend}\;=\;N_{crop}+N_{safety}+N_{loss}-N_{initial}-N_{manure}-N_{min}$$ (1)
  Where N_recommend_ is recommended fertilizer N, N_crop_ is crop N uptake, N_safety_ is soil N_min_ safety margin, N_loss_ is N loss, N_initial_ is soil N_min_ in the root zone before transplanting, and N_manure_ is N_min_ from the mineralization of organic nitrogen in soil.
4. Optimal irrigation with optimal N fertilization (IoNo): Optimal irrigation was applied based on our previous study (for details see the treatment IoNc), and N fertilization was applied at rates based on N balance (Table 1).

**Table 1:** The application amount of chemical fertilizer and irrigation water under different treatments in the autumn-winter (AW) and winter-spring (WS) cropping seasons.

Days after transplanting	Chemical N fertilizer (kg ha^-1^)				Irrigation water (m³ ha^-1^)			
	IoNo	IoNc	IcNo	IcNc	IoNo	IoNc	IcNo	IcNc
AW season								
3	0	0	0	0	525	525	525	525
15	0	0	0	0	525	525	525	525
29	42.5	90	42.5	90	225	225	450	450
50	42.5	90	42.5	90	225	225	450	450
75	42.5	90	42.5	90	225	225	450	450
88	42.5	90	42.5	90	225	225	450	450
Total inputs	170	360	170	360	1950	1950	2850	2850
WS season								
4	0	0	0	0	525	525	525	525
30	50	165	50	165	300	300	450	450
41	50	165	50	165	300	300	450	450
53	50	165	50	165	300	300	450	450
61	50	165	50	165	300	300	450	450
69	50	165	50	165	300	300	450	450
77	50	165	50	165	300	300	450	450
85	50	165	50	165	300	300	450	450
Total inputs	350	1155	350	1155	2625	2625	3675	3675

For each time of N fertilization, all plots received the same chemical P and K inputs. For each time the inputs of P_2_O_5_ and K_2_O were 24.5 and 80.6 kg ha^-1^ in the AW season, and were 20.5 and 68.5 kg ha^-1^ in the WS season, respectively. The total inputs of P_2_O_5_ and K_2_O were 98 and 322.4 kg ha^-1^ in the AW season, and were 143.5 and 480 kg ha^-1^ in the WS season, respectively.

The experiment was a randomized block design with four replications and the size of each replicate plot was 3.6 m × 5.6 m. Each replicate plot had three cultivation furrows and was separated from the adjacent plots by plastic films buried at a depth of 100 cm. For each season, irrigation rates under optimal and conventional treatments were 225 and 450 m³ ha^-1^ in the AW season, and were 300 and 450 m³ ha^-1^ in the WS season, respectively. The chemical compound fertilizer used in this study was a water-soluble fertilizer called Shengdanshu (N-P-K = 19%-8%-27%). In the conventional fertilization treatments, extra N was provided in the form of urea (46% N). All chemical fertilizers were dissolved and applied through the irrigation water.

### Soil properties

To evaluate the migration of soil water and nutrients under different fertigation treatments, soil samples from five cores per subplot were collected five times within a single fertigation cycle during the middle fruit harvest period when daily fruit production was very high in each cropping season. For the WS season, soil were samples on May 8, May 10, May 12, May 14, and May 16. For the AW season, the corresponding sampling times were December 28, December 30, January 2, January 6, and January 10. Soils samples were taken at 0–20, 20–40, 40–60, 60–80 and 80–100 cm depths. Soil samples of each plot at each depth were mixed thoroughly and then passed through a 2-mm sieve. Soil water content was measured drying 20 g fresh soil at 105 °C to constant weight. Soil nitrate and ammonium were extracted using 0.01 M CaCl_2_ solution, and the extracts were analyzed by using a continuous flowing analyzer (TRAACS2000, USA) [28]. Soil available P was analyzed as the method described by Olsen et al. (1954) [29]. Soil available K were extracted using 1mM NH_4_OAc, and the extracts were analyzed by using a flame photometry (Flame Photometer 410, UK).

### Leaching water characteristics

**Fig 1:**
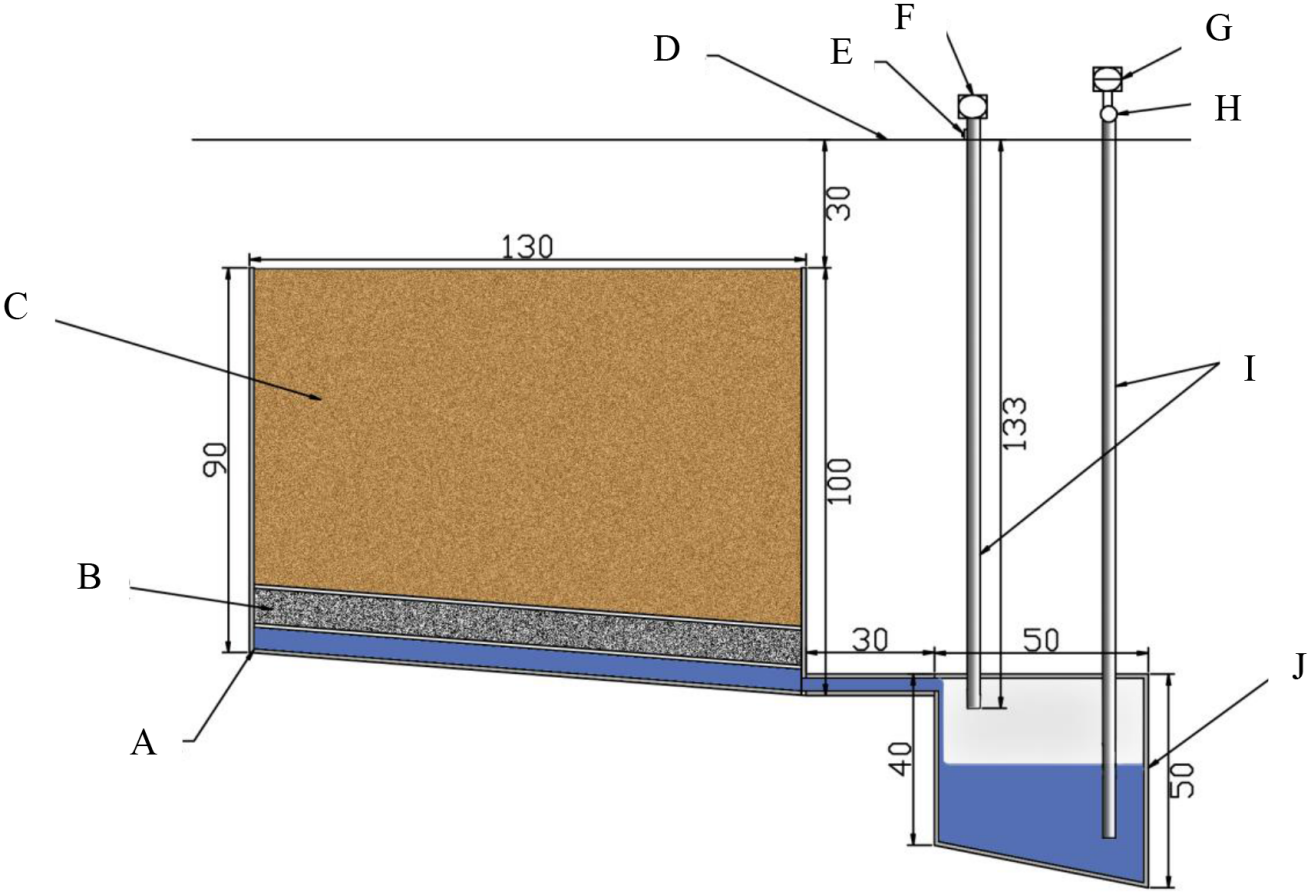
Sketch of the lysimeters. A, box. B, filter layer. C, test Soils. D, soil surface. E, snorkel off valve. F, pressure pump. G, aspirator. H, graduate. I, plastic pipe. J, bucket.

Sixteen drainage lysimeters were installed in each plot, with 150 cm length, 130 cm width, and 90 cm height. The drainage lysimeter occupied an area of 1.95 m^2^, and was installed on top of the soil surface to facilitate farming (Fig 1). During every irrigation cycle, the leached water was collected by using a vacuum pump. The leachate sample was collected in a polyethylene bottle. After measuring the leachate volume, the leachate sample was filtered (0.45 μm) and then stored at 4 °C until the further analysis. Total dissolved P (TDP), K (TDK), Ca (TDCa), Mg (TDMg), Na (TDNa), Fe (TDFe), Mn (TDMn), Cu (TDCu) and Zn (TDZn) were determined by inductively coupled plasma optical emission spectroscopy (ICP-OES; iCAP 6300, Thermo Scientific, USA). The nitrate and ammonium N were determined by continuous flowing analyzer (TRAACS2000, USA).

The amount (mg) of nutrient load leached (NL) was computed as follows:

$$NL\;=\;VT\times C_{e}$$ (2)

where *Ce* is the concentration (mg L^-1^) of any of the nutrient leached elements, *VT* is the total volume of water leached per hectare in each growth cycle (L). The total volume of water leached (*VT*) was determined as follows:

VT=VB×10000/AC (3)

where *VB* is the volume of water pumped from the bucket lysimeter (L), and *AC* is the area of the bucket lysimeter’s catch pan (m^2^).

### Plant parameters

Twelve cucumber plants were sampled in each plot after harvest. Plants were separated into roots, leaves and stems. Fresh plant samples were dried in an oven at 70 °C to constant weight, and then the dry samples were analyzed for total N, P, Ca, Mg, and K. The total N was determined by the Kjeldahl technique [30]. The elements P, Ca, Mg, and K were measured using an inductively coupled plasma spectrometer (iCAP 6300, Thermo Scientific, USA).

### Cucumber fruit yield, irrigation water use efficiency and partial factor productivity of applied nitrogen

Commercial yield was measured for whole cucumber growth cycles in each plot and translated into commercial yield weight per hectare. The ratio of yield to water supply was referred to as irrigation water use efficiency (*IWUE*, kg m^-^³):

IWUE=Y/W (4)

Where *Y* and *W* represent the commercial yield (kg ha^-1^) and the amount of water (m³) applied to the cucumber during the growing cycle, respectively. The ratio of yield to N supply is referred to as partial factor productivity of applied N (*PFP*_N_, kg kg^-1^):

$${PFP}_{N}\;=\;Y/F$$ (5)

Where *F* is the amount of fertilizer N (kg ha^-1^) applied to the cucumber during the growing cycle.

### Statistical analysis

Data were subjected to analysis of variance (ANOVA) carried out by SPSS 19.0. Soil properties and plant parameters were analyzed by three-way ANOVA with the factors irrigation (I), N fertilization (N), cropping season (CS), the I×N, I×CS, N×CS, and I×N×CS interactions. Leachate characteristics were analyzed by three-way ANOVA with the factors irrigation I, N, sampling time (ST), the I×N, I×ST, N×ST, and I×N×ST interactions. When analysis generated a significant F value (*P* < 0.05) for the treatments, the means were compared by least significant difference (LSD) test.

## Results and discussion

### Soil water content, mineral N, available P and available K

**Fig 2:**
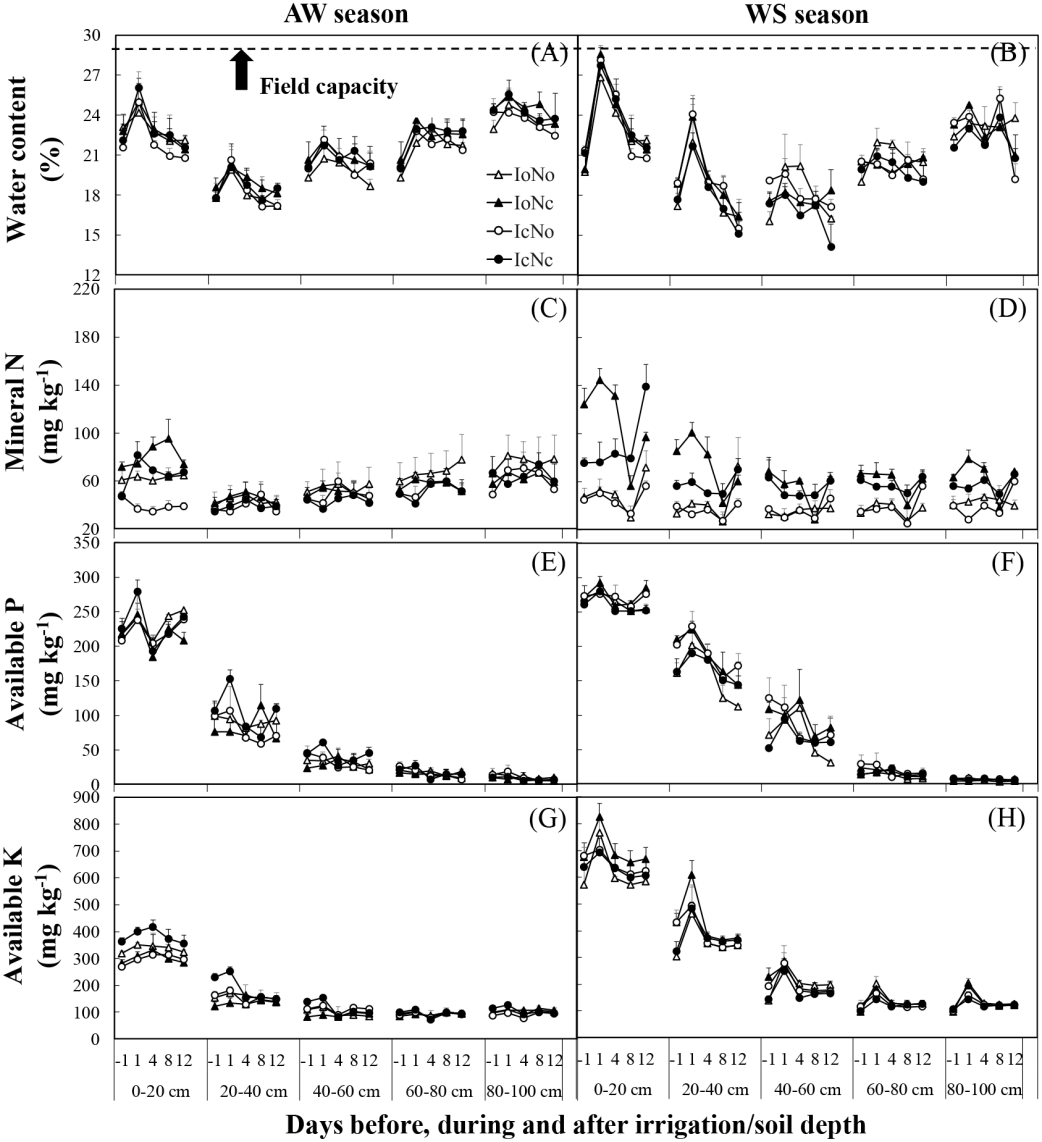
Changes of soil water content (A and B), mineral N (C and D), available P (E and F) and available K (G and H) under different treatments in the autumn-winter (AW) and winter-spring (WS) seasons. Treatments codes are the same as in Table 1. The numbers on the abscissa represent the days before (negative value), during (zero) and after (positive value) irrigation. Bars represent standard errors (n = 4).

The spatial and temporal distribution of water and mineral ions is heterogeneous in soil. Plant growth is generally influenced not only by nutrient concentrations, but also by the spatiotemporal variation of water and nutrients [31,32]. In our experiment, within an irrigation cycle, for all treatments soil water content increased rapidly and then decreased gradually to relatively stable levels in the AW season, and sustainedly decreased in the WS season (Fig 2A and 2B). Both irrigation and fertilization treatments did not statistically influence soil water content in all tested soil layers (Table 2). For the optimal irrigation treatments, the soil water content reached the 95% upper limitation of field capacity that we set. It seems unreasonable since the irrigation amount was obviously higher for the optimal treatments as compared to the conventional treatments (Table 1). However, the volume of leachate occurred in the first 24h was accounted for 94.5% of the total volume of leachate in a simulation experiment [33], hence, this phenomenon could be easily explained because the leaching process of soil water was generally occurred in a few hours after irrigation and for all treatments soil water content after once irrigation exceeded the field capacity (29%; Fig 2) in all tested soil layers in both cropping seasons.

**Table 2:** Analysis of variance of the effects of irrigation (I), nitrogen fertilization (N) and cropping season (CS) on soil properties.

Soil properties	I	N	CS	I×N	I×CS	N×CS	I×N×CS
0-20cm							
Water content	0.02^NS^	0.12^NS^	2.28^NS^	0.09^NS^	0.34^NS^	0.41^NS^	1.41^NS^
Mineral N	14.19**	84.33***	8.24*	1.09^NS^	0.05^NS^	19.26***	1.84^NS^
Available P	0.01^NS^	0.09^NS^	40.24***	0.04^NS^	0.23^NS^	0.02^NS^	3.36^NS^
Available K	0.01^NS^	2.14^NS^	268.31***	0.04^NS^	1.03^NS^	0.04^NS^	7.77*
20-40cm							
Water content	0.00^NS^	0.70^NS^	0.80^NS^	3.62^NS^	0.11^NS^	1.20^NS^	0.56^NS^
Mineral N	5.12*	11.57**	7.91*	0.47^NS^	0.77^NS^	11.34**	0.31^NS^
Available P	0.61^NS^	0.31^NS^	116.40***	0.31^NS^	0.00^NS^	0.09^NS^	7.42*
Available K	0.35^NS^	1.14^NS^	196.06***	0.16^NS^	1.23^NS^	0.56^NS^	4.13^NS^
40-60cm							
Water content	0.03^NS^	0.11^NS^	47.31***	1.98^NS^	0.98^NS^	3.91^NS^	0.04^NS^
Mineral N	0.56^NS^	2.58^NS^	0.70^NS^	0.03^NS^	0.37^NS^	7.21*	0.01^NS^
Available P	0.01^NS^	0.35^NS^	84.18***	2.35^NS^	1.74^NS^	0.01^NS^	9.88**
Available K	0.01^NS^	0.28^NS^	87.96***	0.05^NS^	3.31^NS^	0.13^NS^	0.78^NS^
60-80cm							
Water content	0.06^NS^	0.97^NS^	7.35 *	1.57^NS^	0.00^NS^	3.97^NS^	1.08^NS^
Mineral N	0.85^NS^	2.04^NS^	2.81^NS^	0.04^NS^	0.68^NS^	6.62*	0.46^NS^
Available P	0.95^NS^	0.04^NS^	95.22***	0.22^NS^	0.34^NS^	0.18^NS^	3.09^NS^
Available K	0.96^NS^	0.03^NS^	58.37***	0.01^NS^	1.20^NS^	0.00^NS^	0.09^NS^
80-100cm							
Water content	1.96^NS^	0.47^NS^	20.95***	0.12^NS^	0.55^NS^	4.65*	0.04^NS^
Mineral N	0.75^NS^	1.33^NS^	5.95 *	0.25^NS^	0.02^NS^	3.97^NS^	0.64^NS^
Available P	0.38^NS^	1.42^NS^	8.46*	0.48^NS^	0.34^NS^	1.89^NS^	0.15^NS^
Available K	4.97*	0.71^NS^	67.47***	2.23^NS^	0.77^NS^	2.02^NS^	0.86^NS^

The values shown are *F*-values. NS, not significant.

* *P* < 0.05,

** *P* < 0.01,

*** *P* < 0.001.

**Fig 3:**
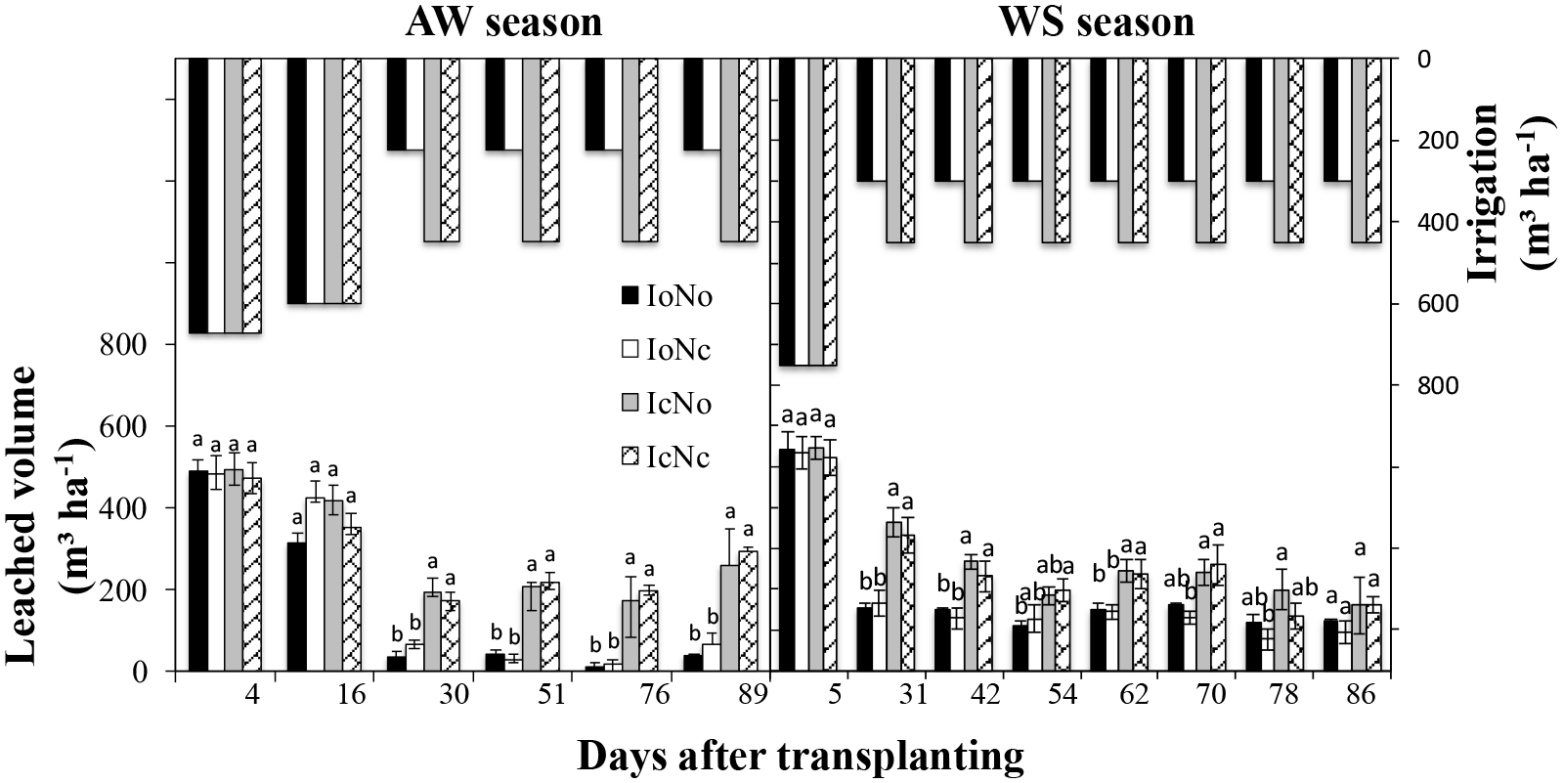
Changes of leached volume on different days after transplanting under different treatments in the autumn-winter (AW) and winter-spring (WS) seasons. Treatments codes are the same as in Table 1. Bars represent standard errors (n = 4). The same letter for each sampling time in the same cropping season indicates no significant difference (P = 0.05).

Soil mineral N in the 0–40 cm layer was significantly influenced by both irrigation and fertilization, and the influence of fertilization was more significant than that of irrigation (*F*_N_>*F*_I_, *P*_N_<*P*_I_; Table 2). This could be easily explained by the higher N input under the IcNc and IoNc treatments as compared to the IcNo and IoNo treatments (Table 1). In general, soil mineral N in the main cucumber root-zone (0–20 cm) [10] was higher under conventional fertilization treatments (IcNc and IoNc) than under optimal fertilization treatments (IcNo and IoNo), especially in the WS season (Fig 2C and 2D). A possible reason is that the soil temperature was overally higher in the WS season than in the AW season [10, 34], resulting in relatively higher soil organic N mineralization and mineral N level in soils in the WS season [35]. Moreover, N requirement by plants was generally higher in the WS season than in the AW season. As a result, relatively higher N input under conventional N fertilization might exceed the N requirement of plants, leading to a relatively higher mineral N in soils in the WS season. We noted that within an irrigation cycle, the fluctuation of soil mineral N in the main root-zone (0–20 cm) was less under optimal fertilization conditions than under conventional fertilization conditions (Fig 2C and 2D). This phenomenon is important because it is essential to maintain a relative stable nutrient supply in cucumber root-zone [10]. However, conventional interval fertigation is still common in intensive vegetable production systems in China. Consequently, the nutrient concentration in soils may exceed plant requirement on the first day after fertigation, and then gradually decrease to reach deficit levels before next fertigation event [36]. Interestingly, optimal irrigation generally increased soil mineral N in the 0–20 cm layer under the same fertilization conditions (compare IoNc versus IcNc, and compare IoNo versus IcNo) at most sampling times within an irrigation cycle, especially under the conventional fertilization (Fig 2C and 2D). This could be due to that the water leaching loss was higher under conventional irrigation conditions than under optimal irrigation conditions (Fig 3), resulting in a relatively higher N leaching to the groundwater under conventional irrigation conditions. Indeed, mineral N and nitrate in particular, is hardly retained in soils and can be easily lost from soils through leaching [9]. Thus, optimal irrigation was more efficient to maintain a relatively high and stable soil mineral N level at root zone and might benefit crop growth.

Soil available P and K were not influenced by both irrigation and fertilization in most tested soil layers (Table 2). One reason is that P and K inputs from fertilizers were equal for all treatments (Table 1). Another reason is probably due to that compared to nitrate N, P and K were more easily retained in soils and relatively hard to lose from soils through leaching [9,37]. Consequently, no marked difference in both soil available P and K was found among treatments (Fig 2E–2H). However, soil available P and K were strongly influenced by cropping season (Table 2). In general, the contents of available P and K were higher in the WS season than in the AS season (Fig 2E–2H). A possible explanation is that microbial activity is more vigorous in the WS season due to the relatively higher soil temperature [10,27], resulting relatively higher organic matter mineralization and available P and K in soils in the WS season [38,39].

### Leaching volume

Leachate volume differed during the various collection periods due to plant transpiration and soil evaporation [17]. In our experiment, under the same irrigation rate, the leachate volume was less in the WS than in the AW season. This is probably due to the fact that the higher average temperature in the WS season led to relatively higher plant transpiration and soil evaporation. Furthermore, solution volume transferred by leaching was positively correlated with the amount of irrigation but not fertilization (Table 3), the optimal irrigation significantly decreased the water leaching loss at most sampling times (Fig 3). In this study, no significant effect of fertilizer rate on leachate volume was found. Similarly, Fare et al. [40] found that the fertilizer treatment had no effect on leachate volume collected from the soil growing *Ilex crenata*. Bayer [41] showed that the leachate volume of L. camara ‘Sunny Side Up’ increased with increasing irrigation duration, regardless of fertilizer rate. As mentioned above, the leaching process of soil water was generally occurred in a few hours after irrigation. Once soil water content exceeded the field capacity (29%) of soils (Fig 2), the excessive water would leach from soils into groundwater (Fig 3). Unsurprisingly, no effect of fertilization on solution volume transferred by leaching was found (Fig 3). It has been demonstrated that increased application of organic fertilizer (manure) could decrease the amount of leachate [37], due to the enhanced water-holding capacity by organic fertilizer. Organic fertilizer application can enhance soil water holding capacity by improving not only physical properties, but also the biological characteristics of soils. In this study, increased application of inorganic fertilizer probably did not influence soil water-holding capacity (Fig 2A and 2B).

**Table 3:** Analysis of variance of the effects of irrigation (I), nitrogen fertilization (N) and sampling time (ST) on cumulative leached amounts of mineral elements in leaching water in the autumn-winter (AW) and winter-spring (WS) cropping seasons.

Leaching water characteristics	I	N	ST	I×N	I×ST	N×ST	I×N×ST
AW season							
Leachate volume	560.1***	0.1^NS^	19.8***	7.1**	9.4***	0.1^NS^	2.1^NS^
NO_3_^-^	228.3***	0.4^NS^	7.6***	4.2*	4.2*	1.8^NS^	3.6*
TDP	63.7***	8.5**	5.4**	3.0^NS^	4.8**	2.6^NS^	3.0*
TDK	179.5***	1.3^NS^	0.5^NS^	0.0^NS^	0.1^NS^	5.2**	7.7***
TDCa	460.1***	0.1^NS^	19.9***	5.8*	10.8***	0.2^NS^	3.5*
TDMg	368.1***	0.7^NS^	12.3***	8.4**	9.3***	2.4^NS^	5.6**
TDNa	312.7***	0.2^NS^	9.2***	1.9^NS^	8.3***	3.2*	5.0**
TDFe	4.7*	4.4*	2.7^NS^	0.4^NS^	2.7^NS^	1.3^NS^	2.3^NS^
TDCu	7.4**	2.2^NS^	0.5^NS^	0.3^NS^	2.0^NS^	0.5^NS^	1.0^NS^
TDZn	18.3***	2.6^NS^	9.4***	7.2**	3.7*	2.0^NS^	2.0^NS^
WS season							
Leachate volume	69.7***	1.1^NS^	2.1^NS^	0.1^NS^	1.1^NS^	0.2^NS^	0.1^NS^
NO_3_^-^	14.3***	1.8^NS^	5.1***	0.8^NS^	1.1^NS^	0.3^NS^	0.2^NS^
TDP	32.0***	0.1^NS^	5.8***	0.9^NS^	4.2**	0.3^NS^	0.1^NS^
TDK	33.3***	0.0^NS^	7.2***	0.1^NS^	0.4^NS^	0.1^NS^	0.1^NS^
TDCa	34.5***	0.1^NS^	6.9***	0.5^NS^	2.4*	0.4^NS^	0.5^NS^
TDMg	11.8**	0.1^NS^	4.9**	0.0^NS^	1.6^NS^	0.2^NS^	0.2^NS^
TDNa	29.5***	1.1^NS^	4.3**	0.0^NS^	1.6^NS^	0.2^NS^	0.3^NS^
TDFe	8.8**	0.2^NS^	2.4*	1.7^NS^	1.4^NS^	1.9^NS^	1.2^NS^
TDCu	14.8***	1.8^NS^	1.8^NS^	0.1^NS^	1.0^NS^	0.6^NS^	1.0^NS^
TDZn	16.6***	0.3^NS^	7.7***	0.1^NS^	1.4^NS^	1.0^NS^	0.8^NS^

The values shown are *F*-values. NS, not significant.

* *P* < 0.05,

** *P* < 0.01,

*** *P* < 0.001.

### The pH, EC and mineral elements in leaching water

**Fig 4:**
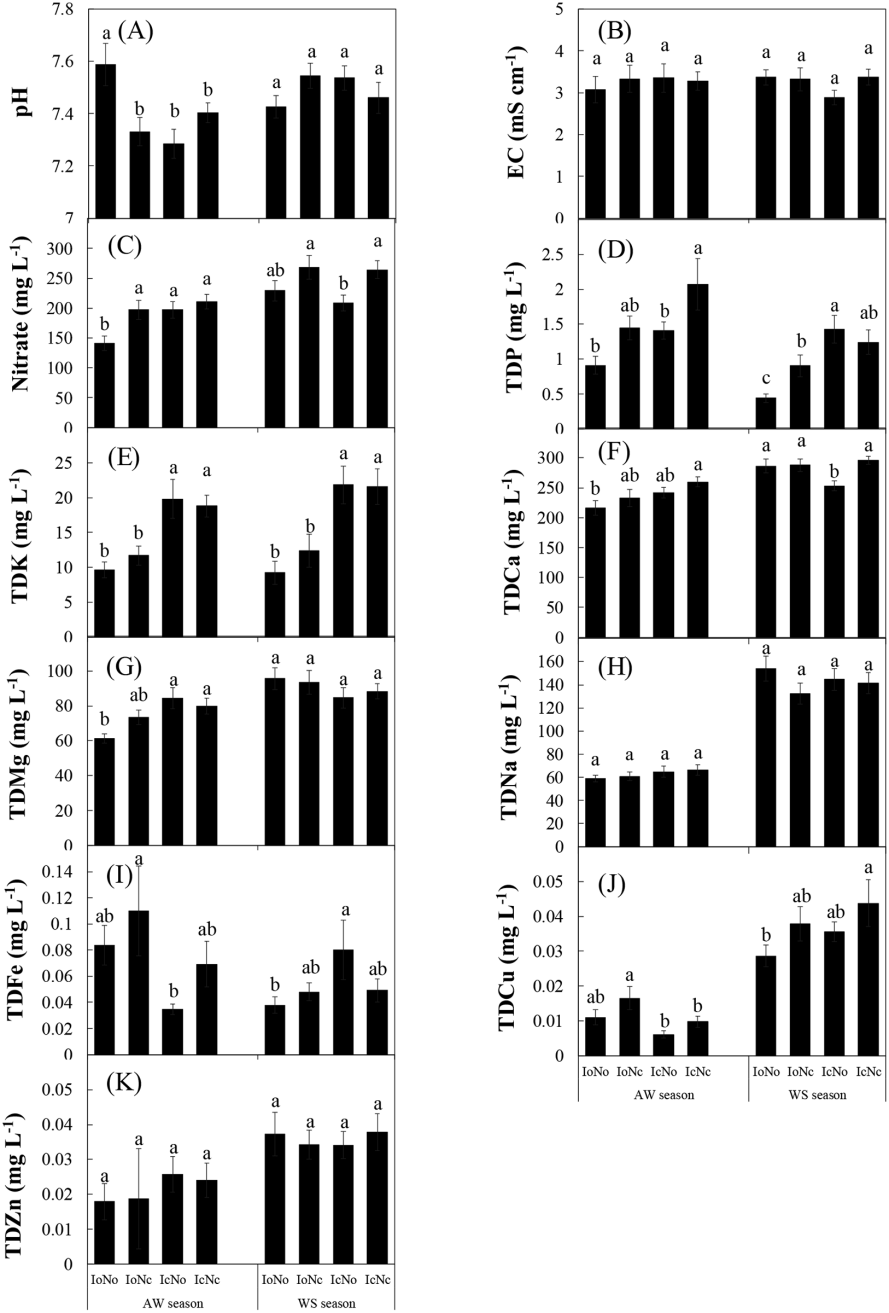
Mineral elements concentrations in leachate under different treatments in the autumn-winter (AW) and winter-spring (WS) seasons. Treatments codes are the same as in Table 1. Bars represent standard errors (n = 4). The same letter for each sampling time in the same copping season indicates no significant difference (P = 0.05).

The pH of leaching water was significantly influenced by irrigation in the AW season (Table 4). However, the effect of irrigation was strongly influenced by fertilization (*P*_I×N_ < 0.001) (Table 4). Thus, it was hard to find a general trend in the pH of leaching water among irrigation treatments in the AW season (Fig 4A). Despite this, a significantly higher pH in leaching water was found under the IoNo treatment as compared to other three treatments on day 30 after transplanting. Similar to the pH, the EC of leaching water was also significantly influenced by irrigation in the AW season, and by the interaction of irrigation and fertilization in the WS season (Table 4). However, only on days 30 and 51 after transplanting, the EC of leaching water was significantly higher under the IcNc treatment than other three treatments (Fig 4B), indicating that the combination of excessive irrigation and excessive fertilization markedly enhanced nutrients and/or salt-related elements leaching. In summary, irrigation had more influence than fertilization on nutrient leaching loss. These results could also be verified in the *Gardenia jasminoides* cultivation system [42].

**Table 4:** Analysis of variance of the effects of irrigation (I) and nitrogen fertilization (N) on concentrations of mineral elements in leaching water in the autumn-winter (AW) and winter-spring (WS) cropping seasons.

Leaching water characteristics	I		N		I×N	
	P value	F value	P value	F value	P value	F value
AW season						
pH	10.8	0.002	0.5	0.483	24.4	<0.001
EC	8.2	0.006	1.3	0.257	1.1	0.304
NO_3_^-^	2.5	0.117	9.8	0.003	0.9	0.347
TDP	8.4	0.006	7.0	0.011	0.4	0.530
TDK	48.4	<0.001	5.4	0.024	0.2	0.696
TDCa	5.9	0.019	1.6	0.207	0.9	0.350
TDMg	12.9	0.001	0.4	0.555	1.2	0.282
TDNa	13.1	0.001	1.2	0.288	0.0	0.849
TDFe	2.0	0.165	2.4	0.131	0.8	0.378
TDCu	4.8	0.033	3.0	0.092	1.6	0.210
TDZn	1.7	0.202	2.5	0.121	2.6	0.113
WS season						
pH	0.1	0.781	0.3	0.614	5.5	0.022
EC	0.2	0.648	0.1	0.771	4.9	0.029
NO_3_^-^	2.0	0.158	5.8	0.019	0.8	0.374
TDP	18.0	<0.001	1.4	0.239	2.4	0.127
TDK	19.0	<0.001	0.9	0.341	0.1	0.790
TDCa	1.6	0.208	2.3	0.136	1.4	0.241
TDMg	2.6	0.110	0.1	0.745	0.3	0.583
TDNa	0.0	0.837	0.4	0.536	0.6	0.438
TDFe	1.8	0.13	0.0	0.913	2.2	0.138
TDCu	2.0	0.165	3.8	0.056	0.0	0.919
TDZn	0.0	0.951	0.0	0.919	0.5	0.314

The nitrate concentration in leaching water was significantly influenced by fertilization in both AW and WS seasons (Table 4). In particular, the lowest nitrate concentration was found under the IoNo treatment on days 4, 16 and 30 after transplanting in the AW season and under the IcNo treatment on days 31, 42, 54, 70 and 78 after transplanting in the WS season, respectively (Fig 4C). Since both the IoNo and IcNo treatments received optimal N inputs, optimal fertilization might be efficient in decreasing the nitrate concentration in leaching water. The benefits of optimal N fertilization have been previously proven in the vegetable productive system [10,43]. However, we noted it was hard to find general trends in the nitrate concentration of leaching water among irrigation and fertilization treatments (Fig 4C). This is probably due to that nitrate leaching from soils can be influenced by several factors, such as plant absorption, microbial mineralization and immobilization, soil temperature and nutrient-holing capacity [9,28].

Concentrations of TDP and TDK in leaching water were significantly influenced by both irrigation and fertilization in the AW season, and by irrigation in the WS season (Table 4). Overally, the effect of irrigation was more significant than that of fertilization (*F*_*I*_ *>F*_*N*_, *P*_*I*_ *< P*_*N*_). Moreover, concentrations of TDCa, TDMg, TDNa, and TDCu in leaching water were significantly influenced by irrigation in the AW season (Table 4). These results suggested that irrigation had more influence than fertilization on leaching water composition in the AW season. Moreover, the soil temperature, soil water evaporation and plant nutrient absorption were generally lower in the WS season than in the AW season [10,27] which may reduce the influence of irrigation on the leaching of Ca, Mg, Na and Cu in in the WS season. Similar to nitrate (Fig 4C), concentrations of TDP, TDK, TDCa, TDMg, TDNa, TDFe, TDCu aand TDZn did not show general trends (Fig 4D–4K).

### Cumulative leaching of water and mineral elements

**Fig 5:**
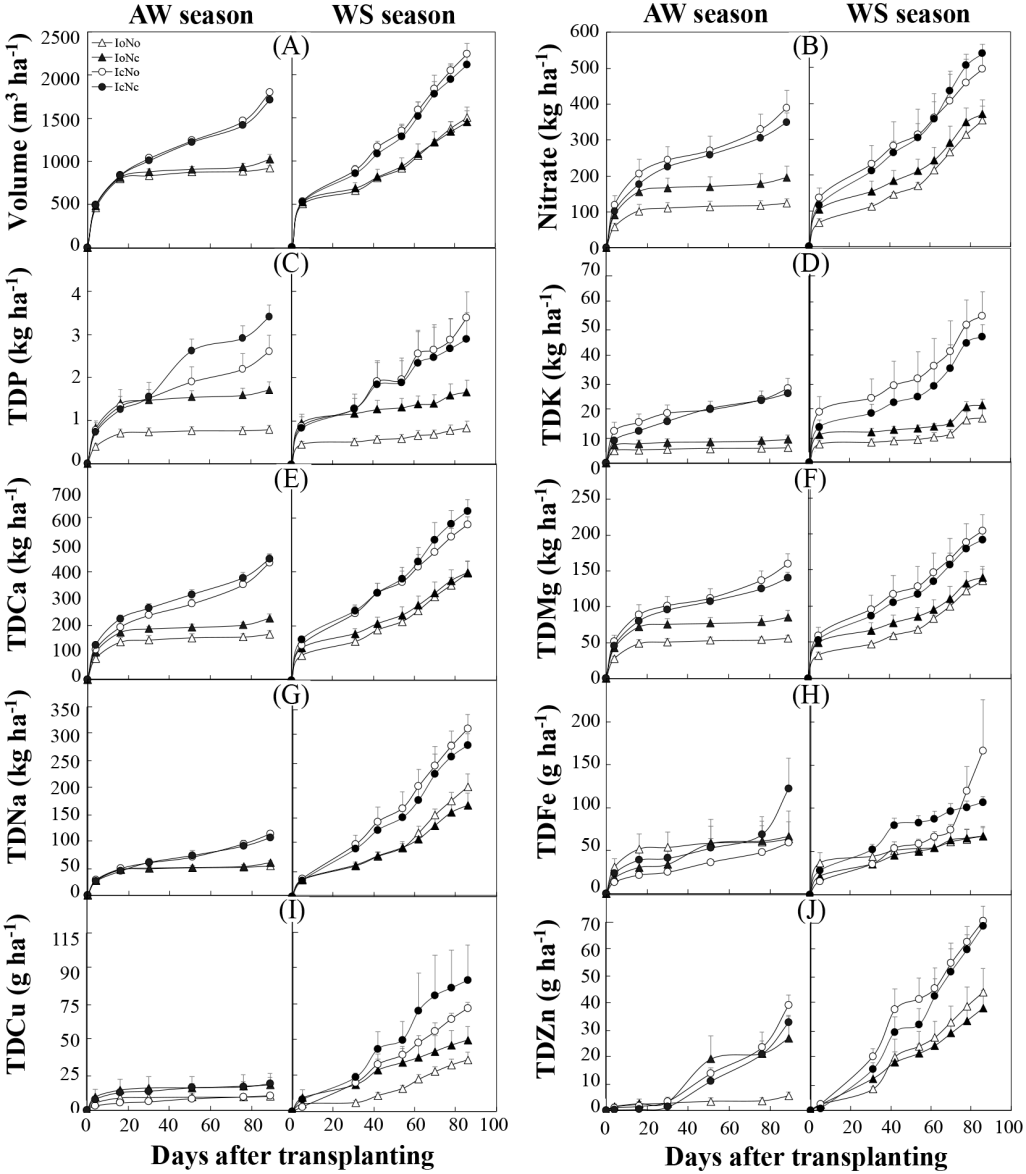
Cumulative leached amounts of water (A), nitrate (B) and total dissolved (TD) mineral elements (B-J) in leaching water under different treatments in the autumn-winter (AW) and winter-spring (WS) seasons. Treatments codes are the same as in Table 1. Bars represent standard errors (n = 4).

The potential environmental risk is strongly influenced by the cumulative leaching of mineral elements. Since the concentration of ammonium was very low and much lower than that of nitrate, and since no significant difference in the concentration of ammonium was found among treatments, only nitrate in leaching water was considered in this study. Generally, cumulative leached amounts of all tested mineral elements were more significantly influenced by irrigation in both AW and WS seasons, and by interaction of irrigation and fertilization in AW season (Table 3). The fertilization only influenced cumulative leached amounts of TDP and TDFe in the AW season. Moreover, the influence of irrigation on TDP and TDFe were more significant than that of fertilization (Table 3). These results suggested that irrigation had more influence than fertilization on the potential environmental risk caused by leaching. Due to the significant leaching under conventional irrigation (Fig 5A), cumulative leached amounts of nitrate were significantly higher under the IcNo and IcNc treatments than under the IoNo and IoNc treatments in both AW and WS seasons (Fig 5B).

Most previous researches mainly concentrated their attention upon the P loss that was induced by erosion and surface run-off, because the P loss through leaching was generally thought to be insignificant [44]. Recently, however, an increasing number of studies have found that there were significant quantities of P in drainage waters collected from the crop production systems. For instance, Fortune [45] investigated four UK field sites and found the annually cumulative loss of P in drainage waters ranged from 0.03–5 kg P ha^-1^ during the years 2001–2002. This result was basically consistent with the TDP loss in our experiment. Compared to the N and P loss, less attention has been paid to the K loss in previous studies. Generally, K can be leached in sandy soil with low clay by rainfall and irrigation water [45]. Alfaro [46] reported that under 480 mm rainfall over a 7-month period, the K loss due to leaching ranged from 1–39 kg ha^-1^. In this study, however, the cumulative loss of total K exceeded 50 kg ha^-1^ over a 3-month period under conventional irrigation treatments in the WS season. It seems that the K loss due to leaching could easily occur in an excessively fertilized soil.

To our knowledge, the leaching loss of P, K, Ca, Mg, Fe, Cu and Zn was generally ignored in previous studies regarding N fertilization. Optimal irrigation also significantly decreased cumulative leached amounts of TDP, TDK, TDCa, TDMg and TDNa in both AW and WS seasons, and TDFe, TDCu and TDZn in the WS season (compare IoNo and IoNc versus IcNo and IcNc; Fig 5C–5J), respectively, further demonstrating the important role played by irrigation in reducing the potential environmental risk caused by leaching. As mentioned above, water is essential for the nutrient leaching process. More importantly, since these nutrients were not leached under optimal irrigation, nutrients remain in the soil and could be used by the plant. Interestingly, under optimal irrigation, conventional N fertilization significantly increased the cumulative leached amount of TDP in both AW and WS seasons (compare IoNc versus IoNo; Fig 5C). One possible reason is that higher nitrate might increase soil microbial mineralization, thereby increasing mineral P in soils and enhancing P leaching [47, 48]

**Fig 6:**
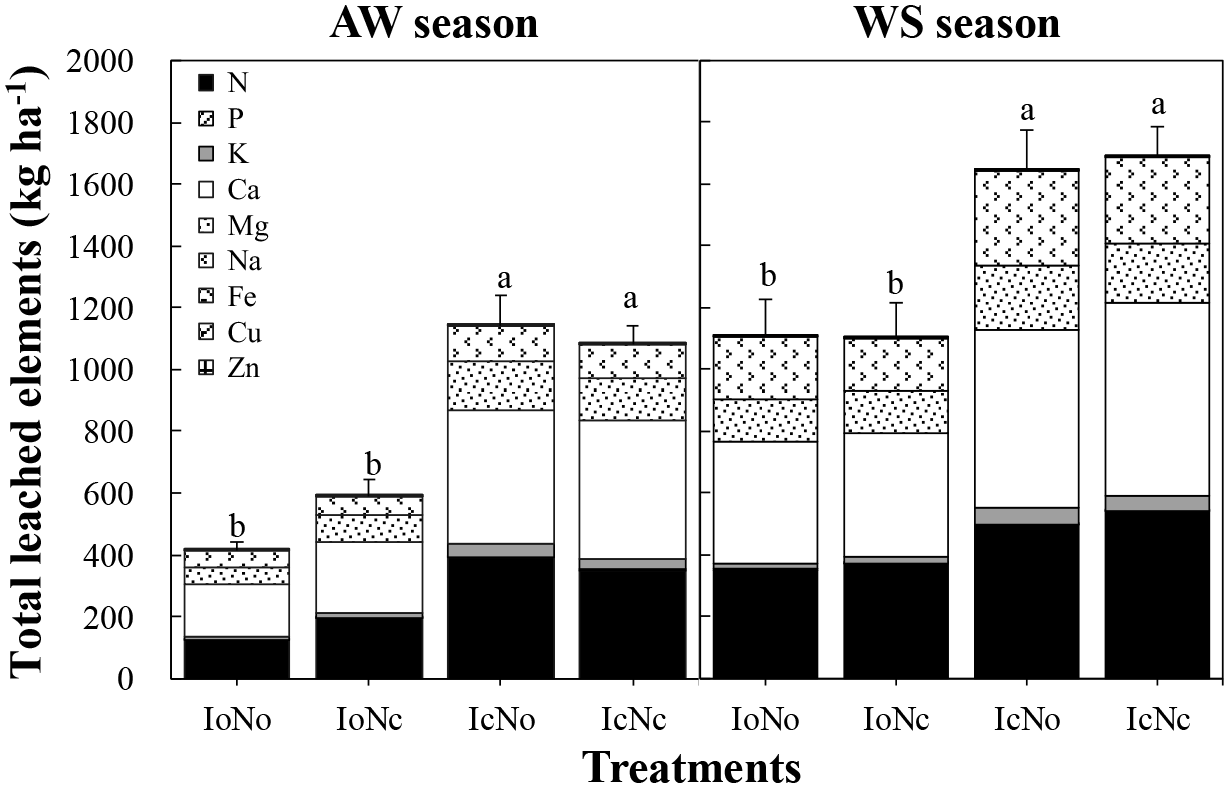
The total leached amounts of mineral elements under different treatments in the autumn-winter (AW) and winter-spring (WS) seasons. Treatments codes are the same as in Table 1. Bars represent standard errors (n = 4). The same letter in the same cropping season indicates no significant difference (P = 0.05).

Overally, the total leached amounts of most tested elements were significantly influenced by irrigation but not fertilization (Table 3). Only TDP and TDFe were significantly influenced by fertilization in the AW season. In addition, the interaction of irrigation and fertilization significantly influenced NO_3_^-^, TDCa, TDMg and TDZn in the AW season. In specific, the total leached amount was decreased by optimal irrigation but not optimal fertilization (Fig 6).

**Fig 7:**
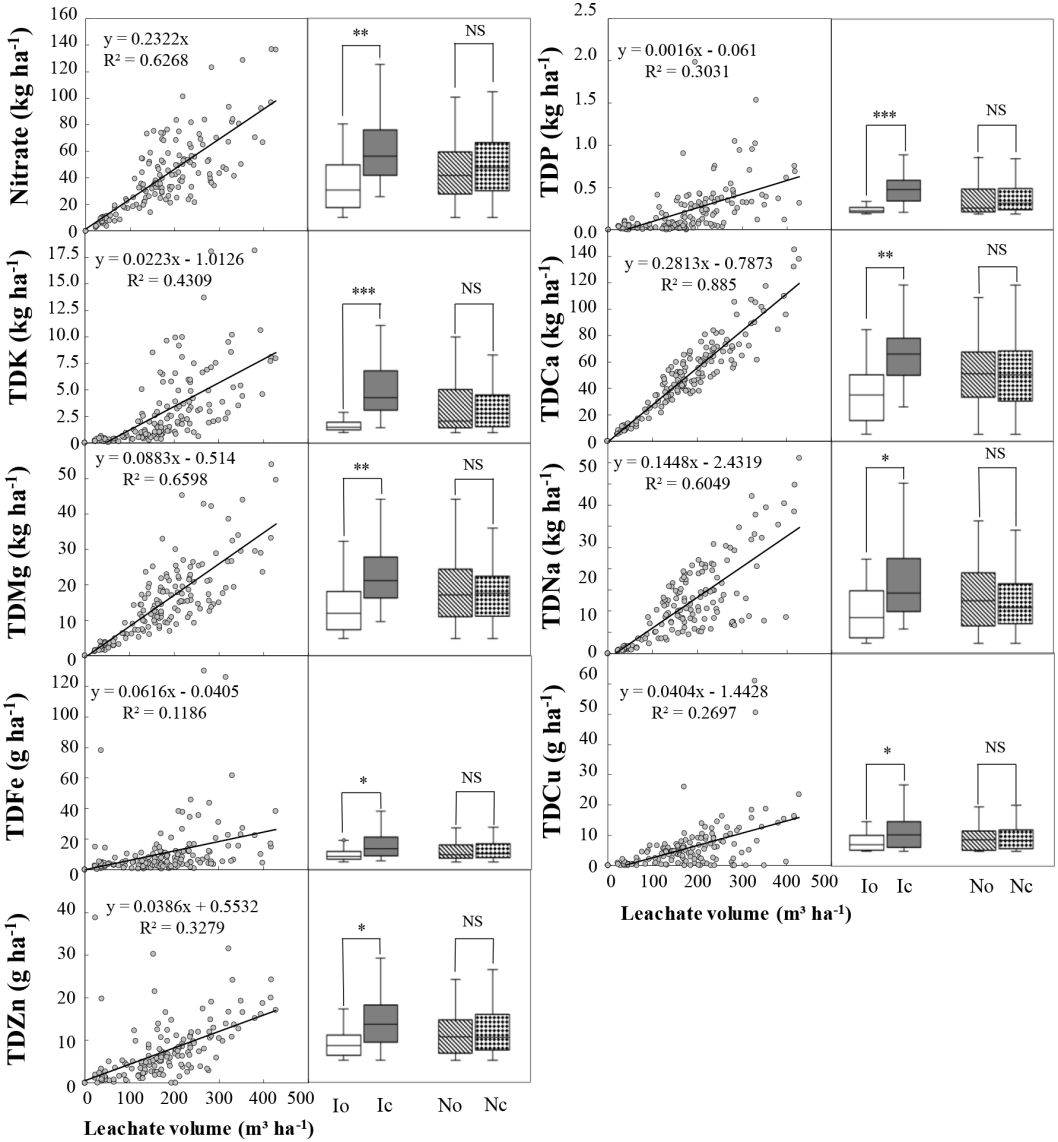
Relationships between the leachate volume and cumulative leached amounts of nitrate and total dissolved (TD) mineral elements in leaching water (n = 156). Treatments codes are the same as in Table 1. * P < 0.05; **P < 0.01; *** P < 0.001; NS: not significant.

In addition, for all tested elements, strongly positive relationships were found between the leachate volume and cumulative leached amount, and these relationships were obviously influenced by irrigation (compare IoNc versus IcNc, compare IoNo versus IcNo) but not fertilization (compare IoNo versus IoNc, compare IcNo versus IcNc; Fig 7). These results further demonstrated the importance1 of irrigation management in intensive vegetable production system.

Based on general knowledge, under the same irrigation, excessive fertilization should have higher nutrient leaching and more influence on leaching water quality than optimal fertilization. In our intensive greenhouse vegetable production systems, however, we surprisingly found that under the same irrigation condition, generally no influence of fertilization on total nutrient leaching and leaching water quality was found (Figs 6 and 7). Moreover, under the same fertilization condition, excessive irrigation strongly increased nutrient leaching and influenced leaching water quality. This means irrigation must be paid more attention during fertilization management in Chinese intensive greenhouse vegetable production systems.

### Nutrient elements in shoots and roots

Generally, effects of irrigation and fertilization on most mineral elements in shoots and roots were not significant (Table 5). However, total N in roots was significantly influenced by irrigation, and total P in roots was significantly influenced by both irrigation and fertilization. Specifically, only total P in the root was significantly higher under the IoNc treatment than under the IcNo treatment (Table 6), suggesting the slight influence of conventional irrigation and fertilization on nutrient absorption by plants. With respect to micronutrients in plant tissues, there was no significant influence by irrigation and fertilization. The microelement plays a very important role in plant growth at both the physiological and molecular levels, however, plants could maintain a relatively stable microelement content unless under severe growth conditions [49].

**Table 5:** Analysis of variance of the effects of irrigation (I), nitrogen fertilization (N) and cropping season (CS) on plant parameters.

Plant parameters	I	N	CS	I×N	I×CS	N×CS	I×N×CS
Root							
Total N	4.90*	0.20^NS^	21.25***	0.08^NS^	0.02^NS^	0.42^NS^	0.12^NS^
Total P	4.91*	8.96**	28.23***	0.87^NS^	0.05^NS^	0.58^NS^	2.01^NS^
Total K	0.40^NS^	0.27^NS^	1.88^NS^	0.04^NS^	2.67^NS^	0.14^NS^	1.70^NS^
Total Ca	0.56^NS^	1.19^NS^	21.05***	1.30^NS^	1.20^NS^	0.03^NS^	0.03^NS^
Total Mg	0.21^NS^	3.02^NS^	7.34*	0.04^NS^	0.44^NS^	0.01^NS^	0.49^NS^
Total Na	1.20^NS^	0.54^NS^	38.89***	1.99^NS^	2.12^NS^	1.42^NS^	0.02^NS^
Total Fe	0.38^NS^	1.75^NS^	0.93^NS^	0.47^NS^	0.26^NS^	0.03^NS^	0.08^NS^
Total Mn	0.00^NS^	0.07^NS^	1.02^NS^	0.66^NS^	1.24^NS^	0.73^NS^	0.15^NS^
Total Cu	0.72^NS^	0.03^NS^	1.38^NS^	0.56^NS^	1.01^NS^	1.29^NS^	1.60^NS^
Total Zn	0.38^NS^	0.35^NS^	0.34^NS^	1.26^NS^	1.84^NS^	0.14^NS^	0.49^NS^
Shoot							
Total N	1.72^NS^	0.00^NS^	1.72^NS^	0.90^NS^	8.12**	1.60^NS^	0.74^NS^
Total P	0.01^NS^	0.86^NS^	14.27***	0.49^NS^	0.80^NS^	1.33^NS^	4.54*
Total K	0.05^NS^	1.92^NS^	244.05***	0.66^NS^	5.30*	0.62^NS^	0.15^NS^
Total Ca	1.59^NS^	2.63^NS^	1.54^NS^	0.05^NS^	1.18^NS^	1.84^NS^	0.04^NS^
Total Mg	0.00^NS^	0.00^NS^	0.03^NS^	0.01^NS^	0.03^NS^	0.74^NS^	0.08^NS^
Total Na	0.06^NS^	0.01^NS^	48.64***	4.27^NS^	0.36^NS^	0.05^NS^	4.68*
Total Fe	3.64^NS^	0.00^NS^	12.42***	0.05^NS^	2.32^NS^	1.33^NS^	2.29^NS^
Total Mn	3.64^NS^	0.78^NS^	0.96^NS^	0.21^NS^	1.77^NS^	1.71^NS^	2.99^NS^
Total Cu	0.02^NS^	0.08^NS^	17.89***	0.98^NS^	1.27^NS^	1.90^NS^	0.03^NS^
Total Zn	1.13^NS^	0.89^NS^	1.34^NS^	1.01^NS^	1.13^NS^	0.77^NS^	1.03^NS^
Yield and utilization efficiency							
Root biomass	0.07^NS^	0.03^NS^	9.59**	0.01^NS^	0.14^NS^	0.84^NS^	1.1^NS^
Shoot biomass	1.01^NS^	1.01^NS^	1.10^NS^	0.95^NS^	0.97^NS^	0.99^NS^	1.07^NS^
Fruit yield	0.00^NS^	1.21^NS^	240.02***	0.25^NS^	0.11^NS^	2.32^NS^	0.01^NS^
IWUE	84.85***	1.69^NS^	82.49***	0.08^NS^	1.88^NS^	3.17^NS^	0.13^NS^
PPF_N_	0.01^NS^	405.68***	24.52***	0.19^NS^	0.10^NS^	13.56**	0.00^NS^

The values shown are *F*-values. NS, not significant.

* *P* < 0.05,

** *P* < 0.01,

*** *P* < 0.001.

**Table 6:** Concentration of macro- and micro-elements in roots and shoots under different treatments in autumn-winter (AW) and winter-spring (WS) cropping seasons. Treatments codes are the same as in Table 1.

Cropping season		Treatments	N (mg g^-1^)	P (mg g^-1^)	K (mg g^-1^)	Ca (mg g^-1^)	Mg (mg g^-1^)	Na (mg g^-1^)	Fe (mg g^-1^)	Mn (mg g^-1^)	Cu (mg g^-1^)	Zn (mg g^-1^)
Roots	AW	IoNo	28.4±1.5a	9.1±0.4ab	45.7±1.7a	10.4±0.1a	5.58±0.24a	8.40±0.07a	2.11±0.25a	0.06±0.01a	0.03±0.01a	0.10±0.01a
		IoNc	29.2±1.9a	10.3±0.4a	46.1±2.3a	11.9±1.1a	5.37±0.32a	8.68±1.33a	1.88±0.27a	0.58±0.01a	0.02±0.01a	0.15±0.04a
		IcNo	23.1±3.1a	8.3±0.4b	43.5±1.7a	11.5±0.7a	5.52±0.47a	6.69±0.16a	2.98±0.92a	0.78±0.02a	0.03±0.01a	0.17±0.05a
		IcNc	26.1±1.5a	9.7±0.4ab	39.7±2.8a	11.3±0.7a	4.81±0.03a	8.12±0.49a	1.95±0.16a	0.64±0.00a	0.02±0.01a	0.15±0.01a
	WS	IoNo	20.5±0.6a	10.8±0.8a	46.9±2.7a	14.3±0.5a	4.87±0.36a	5.52±0.74a	2.77±0.39a	0.07±0.01a	0.03±0.01a	0.13±0.01a
		IoNc	20.3±4.9a	12.4±0.1a	43.8±3.5a	15.8±0.8a	4.23±0.35a	4.66±0.51a	2.46±0.17a	0.09±0.02a	0.03±0.01a	0.15±0.01a
		IcNo	17.0±1.6a	10.8±0.3a	46.0±2.8a	13.7±1.5a	4.79±0.65a	5.03±0.43a	2.99±1.17a	0.07±0.01a	0.02±0.01a	0.13±0.01a
		IcNc	16.6±1.9a	10.7±0.5a	48.5±2.9a	13.9±1.3a	4.42±0.40a	5.51±0.42a	2.33±0.52a	0.06±0.01a	0.03±0.01a	0.13±0.01a
Shoots	AWW	IoNo	31.9±1.4a	10.7±1.0a	44.1±0.6a	48.3±4.1a	15.51±1.15a	1.31±0.13a	1.02±0.17a	0.07±0.01a	0.07±0.02a	0.05±0.01a
		IoNc	33.3±0.8a	12.1±1.0a	42.9±2.2a	48.9±0.1a	14.62±0.35a	1.02±0.14a	0.51±0.09a	0.06±0.01a	0.05±0.01a	0.07±0.01a
		IcNo	32.6±0.7a	9.9±1.4a	41.1±2.2a	47.7±1.5a	15.41±0.41a	0.93±0.02a	0.63±0.16a	0.06±0.01a	0.06±0.01a	0.43±0.36a
		IcNc	24.6±1.3a	12.8±0.3a	41.0±0.8a	48.5±5.1a	15.01±0.98a	1.28±0.15a	0.75±0.16a	0.07±0.01a	0.05±0.01a	0.08±0.01a
	WS	IoNo	26.8±6.2a	7.6±0.0a	24.2±2.4a	51.5±1.5a	14.92±0.72a	0.59±0.09a	1.46±0.42a	0.07±0.01a	0.03±0.01a	0.06±0.01a
		IoNc	30.3±3.0a	8.4±0.3a	20.2±1.7a	58.5±4.5a	15.81±1.21a	0.59±0.11a	1.91±0.47a	0.09±0.02a	0.02±0.01a	0.05±0.01a
		IcNo	37.8±6.2a	9.0±0.6a	25.8±1.4a	43.9±4.4a	14.92±1.36a	0.63±0.07a	1.07±0.12a	0.06±0.01a	0.02±0.01a	0.06±0.01a
		IcNc	40.8±3.7a	8.0±0.3a	24.6±0.4a	53.2±5.5a	15.51±1.94a	0.61±0.05a	1.05±0.18a	0.06±0.01a	0.04±0.01a	0.05±0.01a

Values are mean ± SE (n = 4). The same letter in the same data column for roots and shoots in the same cropping season indicates no significant difference (*P* = 0.05).

### Plant biomass, fruit yields, IWUE and PFP_N_

**Fig 8:**
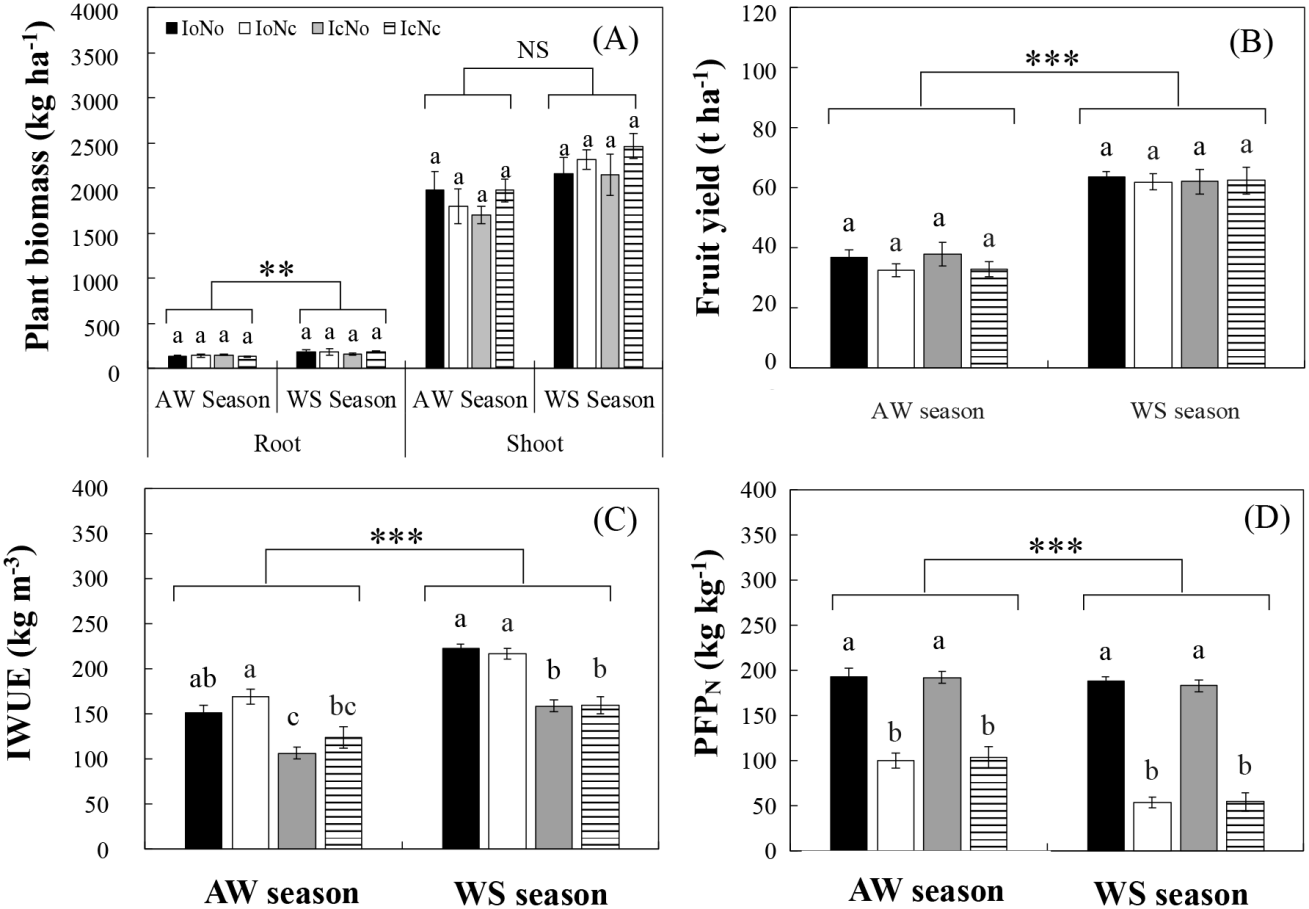
Plant biomass (A), fruit yield (B), irrigation water use efficiency (IWUE, C) and partial factor productivity of nitrogen (PFP_N_, D) under different treatments in the autumn-winter (AW) and winter-spring (WS) seasons. Treatments codes are the same as in Table 1. Bars represent standard errors (n = 4). The same letter in the same cropping season indicates no significant difference (A, C, D), and the same letter in the two cropping season indicates no significant difference (P = 0.05). ** P < 0.01, *** P < 0.001, NS: not significant.

Due to the similar nutrient and water levels in the root-zone soils in, there were no significant difference among treatments for both plant biomass and cucumber fruit yield in same cropping season (Fig 8A and 8B). However, because the WS season had a higher average temperature (23.3 °C) compared to the AW season (13.4 °C), all treatments showed higher plant biomass and fruit yield in the WS season than in the AW season. However, the IWUE was significantly increased by optimal irrigation (compare IoNo versus IcNo, compare IoNo versus IcNc; Fig 8C), while the PFP_N_ was significantly increased by optimal fertilization (compare IoNo versus IoNc, compare IcNo versus IcNc; Fig 8D). In particular, the IoNo treatment increased PFP_N_ by 87.3% in the AW season, and IWUE, PFP_N_ by 40.7% and 249% in the WS season, respectively, when compared to the IcNc. This finding strongly suggests that the most effective strategy to reduce nutrients is to encourage farmers applying the appropriate amount of water and fertilizer, especially for the water. In addition, the application time and method of fertigation need to be considered and researched in future studies.

## Conclusions

Excessively fertilized vegetable soils generally had high nutrient leaching. Optimal irrigation was more efficient than optimal fertilization in reducing nutrient leaching in excessively fertilized soils. In addition, irrigation had more influence than fertilization on leaching water quality in excessively fertilized soils. In general, fertilization merely influenced concentrations of nitrate (NO_3_^-^), phosphorus (P) and potassium (K), but did not affect most leaching water characteristics. In contrast, irrigation influenced pH, EC and concentrations of P, K, Ca, Mg, Na and Cu. Cumulative leached amounts of NO_3_^-^, P, K, Ca, Mg, Na, Fe, Cu and Zn were significantly decreased by optimal irrigation as compared to conventional irrigation under same fertilization conditions, but not by optimal fertilization as compared to conventional fertilization under same irrigation conditions.
